# Circulating microRNAs in Symptomatic and Asymptomatic Carotid Stenosis

**DOI:** 10.3389/fneur.2021.755827

**Published:** 2021-11-24

**Authors:** Gerrit M. Grosse, Anselm A. Derda, Ricarda D. Stauss, Lavinia Neubert, Danny D. Jonigk, Mark P. Kühnel, Maria M. Gabriel, Ramona Schuppner, Mathias Wilhelmi, Christian Bär, Johann Bauersachs, Claudia Schrimpf, Thomas Thum, Karin Weissenborn

**Affiliations:** ^1^Department of Neurology, Hannover Medical School, Hannover, Germany; ^2^Department of Cardiology and Angiology, Hannover Medical School, Hannover, Germany; ^3^Institute of Molecular and Translational Therapeutic Strategies (IMTTS), Hannover Medical School, Hannover, Germany; ^4^Institute of Pathology, Hannover Medical School, Hannover, Germany; ^5^Department of Vascular- and Endovascular Surgery, St. Bernward Hospital, Hildesheim, Germany; ^6^Division of Vascular and Endovascular Surgery, Department of Cardiothoracic-, Transplantation- and Vascular Surgery, Hannover Medical School, Hannover, Germany; ^7^Rebirth Center for Translational Regenerative Therapies, Hannover Medical School, Hannover, Germany; ^8^Department of Vascular Surgery, University Hospital Zurich, Zurich, Switzerland; ^9^Fraunhofer Institute of Toxicology and Experimental Medicine, Hannover, Germany

**Keywords:** atherosclerosis, biomarker, carotid stenosis, microRNA, stroke

## Abstract

**Background:** Specific microRNAs (miRs) have been implicated in the pathophysiology of atherosclerosis and may represent interesting diagnostic and therapeutic targets in carotid stenosis. We hypothesized that the levels of specific circulating miRs are altered in patients with symptomatic carotid stenosis (sCS) in comparison to those in patients with asymptomatic carotid stenosis (aCS) planned to undergo carotid endarterectomy (CEA). We also studied whether miR levels are associated with plaque vulnerability and stability over time after CEA.

**Methods:** Circulating levels of vascular-enriched miR-92a, miR-126, miR-143, miR-145, miR-155, miR-210, miR-221, miR-222, and miR-342-3p were determined in 21 patients with sCS and 23 patients with aCS before CEA and at a 90-day follow-up. Transcranial Doppler ultrasound for detection of microembolic signals (MES) in the ipsilateral middle cerebral artery was performed prior to CEA. Carotid plaques were histologically analyzed.

**Results:** Mean levels of miRs were not considerably different between groups and were only marginally higher in sCS than aCS concerning miR-92a, miR-210, miR-145, and miR-143 with the best evidence concerning miR-92a. After adjustment for vascular risk factors and statin pre-treatment, the effect sizes remained essentially unchanged. At follow-up, however, these modest differences remained uncorroborated. There were no relevant associations between miR-levels and MES or histological plaque vulnerability features.

**Conclusions:** This study does not provide evidence for strong associations between specific circulating miRs and symptomatic state in a collective of comprehensively characterized patients with carotid stenosis. Further work is needed to elucidate the role of circulating miRs as targets in advanced carotid atherosclerosis.

## Introduction

Carotid stenosis is the cause of ischemic stroke or transient ischemic attack (TIA) in about 10–15% of all cases (1). Carotid endarterectomy (CEA) and carotid artery stenting (CAS) are established therapies in secondary stroke prevention in selected patients with symptomatic carotid stenosis (sCS). However, decision-making in patients with asymptomatic carotid stenosis (aCS) is more challenging, and reliable biomarkers identifying patients at high risk of stroke due to carotid stenosis are still lacking.

Recently, novel therapeutic strategies in atherosclerosis have been emerging, mainly addressing inflammatory targets such as Interleukin (IL) 1β (2). Moreover, distinct microRNAs (miRs) have been identified as effectors of functional relevance in atherosclerosis (3, 4) and platelet activation (5, 6). miRs are short non-coding ribonucleic acids that are of interest in various settings of not only novel diagnostic but also therapeutic approaches (7, 8).

Meanwhile, there are various experimental and clinical studies that shed light on expression levels of tissue- and blood-based miRs in patients with and without atherosclerotic disease (9), and a role of certain miRs in the pathophysiology of atherosclerosis and also in the vulnerability of atherosclerotic lesions, e.g., of the carotid artery, has been established (3, 10). The involvement of miRs could be identified, for example, in endothelial injury and inflammation, monocyte, and macrophage differentiation and activation but also in neoangiogenesis, which may contribute to intraplaque hemorrhage within atherosclerotic plaques and enhance the risk of plaque rupture (3, 9).

Of note, most of the studies investigating circulating miRs in human vascular diseases made use of a control group consisting of non-diseased individuals. This study aimed to investigate the levels of distinct circulating miRs implicated in atherosclerosis and platelet activation in patients with advanced atherosclerotic disease, i.e., in sCS vs. aCS. We hypothesized the following: (1) The levels of distinct circulating miRs are higher in patients with sCS than in those with aCS. (2) The levels of circulating miRs are stable over time and still different between sCS and aCS 90 days after enrollment. (3) Circulating miRs are associated with features of plaque vulnerability. Based on the pathophysiology of carotid stenosis, we focused primarily on miRs associated with atherosclerotic disease and, in particular, with inflammation, endothelial, and smooth muscle cell pathology, as well as with platelet activation.

## Materials and Methods

### Study Population and Clinical Data

In this prospective case-control study, we considered patients with sCS or aCS who have been previously described in a previous work from our group investigating distinct plasma cytokine patterns (11). The study recruitment took place between July 2017 and June 2018 at the Departments of Neurology and Cardiothoracic-, Transplantation- and Vascular Surgery at Hannover Medical School. Symptomatic carotid stenoses were identified in patients who suffered an ischemic stroke, a transient ischemic attack (TIA), amaurosis fugax, or central retinal artery occlusion in the corresponding vascular territory. Concurrent stroke etiologies were excluded in patients with sCS in the course of stroke diagnostics including cranial CT (CCT) and/or MRI, CT-, or MR-angiography, transthoracic or transesophageal echocardiography, cardiac rhythm monitoring, and Doppler and duplex ultrasound. Stroke severity was graded using the National Institutes of Health Stroke Scale (NIHSS). In both, sCS and aCS patients with current malignant diseases, acute infectious diseases, immunological diseases, and immunosuppressive therapies were defined as exclusion criteria.

All patients provided written informed consent. The study was approved by the ethics committee at Hannover Medical School (Ethics vote no. 7484-2017) and was conducted according to the ethical principles of the Declaration of Helsinki.

### Study Procedures

We conducted a standardized interview to collect demographical and clinical data as well as current medication. The vascular risk factors were subsumed using the Essen Stroke Risk Score (ESRS). The degree of carotid stenosis was defined according the North American Symptomatic Carotid Endarterectomy Trial (NASCET) criteria and categorized into moderate (50–69%), severe (70–90%), or highly severe (>90%). Long-term clinical outcome was assessed at 3 months after surgery in a follow-up including collection of the NIHSS and the modified Rankin scale (mRS).

In patients with sufficient transtemporal window, we performed a standardized transcranial Doppler (TCD) monitoring of the middle cerebral artery (MCA) on the side of carotid stenosis before surgery. The MCA was insonated in 45–55 mm depth using a 2-MHz transducer and monitoring was recorded for up to 1 h. Microembolic signals (MES) were quantified visually and audibly by an investigator blinded to the clinical data. The detection of MES was performed according to the criteria of the “Consensus on Microembolus Detection” (12).

### Histological Analysis

In the course of CEA, carotid plaques from all patients were obtained and were fixed in 4% buffered formalin immediately after removal. Specimens were embedded in paraffin and 2-μm-thick sections were cut followed by histological staining using Hematoxylin and Eosin, Elastica van Gieson (EvG), and Periodic Acid Schiff (PAS). Histological analysis was done blinded to the clinical characteristics and biomarker results at the Institute of Pathology at Hannover Medical School. Representative sections with maximal atherosclerosis were chosen. Histological examination and staging were performed. The examination included intima fibrosis (divided in stages I–III), lipid core (stages 0–II), calcification (stages 0–III), media degradation (stages 0–III), acute and chronic inflammation (stages 0–III), cholesterol crystals (present/not present), neovascularization (present/not present), intra-plaque hemorrhage (present/not present), fibrous cap rupture (present/not present), and prevalence of an adherent thrombus (present/not present). An amount of points was given for each criterion and summarized to a final score (stages 0–3 resulted in 0–3 points; present/not present led to 1–0 points).

### Biomarker Measurements

Peripheral venous blood was drawn directly prior to CEA as well as at follow-up 3 months later. Full blood was centrifuged at 3,000 g for 15 min and the plasma was stored at −80°C. To isolate RNA, 100 μl plasma was taken and processed with the miRNeasy Serum/Plasma Advanced Kit (Qiagen, Germany) according to the instructions of the manufacturer. This process synthetic cel-miR-39-3p (1.6 × 10^8^ copies/μl) was spiked-in due to normalization in ensuing analysis. To avoid interferences from heparin traces with qPCR reactions, the isolated RNA was treated with heparinase in order to exclude contamination in patients who have been treated with heparin in the hospital (8). Afterwards, the RNA was reverse transcribed to complementary DNA (cDNA) applying the TaqMan MicroRNA Reverse Transcription Kit (Applied Biosystems, MA, USA) as instructed. For amplification, RT-qPCR was implemented using the specific TaqMan miR assays (Applied Biosystems) for hsa-miR-92a, hsa-miR-126, hsa-miR-143, hsa-miR-145, hsa-miR-155, hsa-miR-210, hsa-miR-221, hsa-miR-222, hsa-miR-342-3p, and Cel-miR-39 and a ViiA7 System (Thermo Fisher Scientific, MA, USA).

The acquisition and analyses of clinical data, histology, and biomarker measurements were performed blinded to the group assignments.

### Statistical Analysis

Since this is the first study investigating circulating miRs in patients with symptomatic or asymptomatic carotid stenosis and, accordingly, no previous data on miR levels in these groups were available, an appropriate sample size calculation could not be performed. However, under the hypothesis that distinct inflammatory miRs might reveal a comparable discriminative ability such as, e.g., IL-1β, based on the results deriving from the previous study (11), defining α at 0.05 and the statistical power at 0.80, a sample size of *n* = 22 per group was calculated to reproduce this finding. Thus, it was anticipated that the sample size of this study is appropriate to detect according biomarker differences between the groups.

Relative miR expression levels were calculated and normalized to Cel-miR-39 with the 2^−*dCq*^ method (dCq = Cq[miR] – Cq[Cel-miR-39]). Afterwards, the miR expression data were log2 transformed.

As appropriate, group differences of continuous data were analyzed using the two-sided Student's *t*-test for normally distributed data or the Mann–Whitney *U*-test for ordinal or non-normally distributed data. Categorical data were analyzed using the chi-square test or Fisher's exact test. Correlations of biomarkers with the ESRS were calculated using Spearman's correlation. Binary logistic regression analysis was done by estimating the association of miRs with the study group adjusting for ESRS and statin pre-treatment, as these were relevant confounders.

Group differences were visualized using the Gardner-Altman estimation plots.

Statistical analyses were conducted using the IBM SPSS Statistics 26 (IBM SPSS, Armonk, NY, USA) and the SAS Enterprise Guide 7.1 (SAS Institute Inc., Cary, NC, USA). Figures were created using the GraphPad Prism 9.0.1 (GraphPad Inc., La Jolla, CA, USA).

## Results

Out of 50 patients originally included in the study, four patients were excluded (i.e., one withdrawal of informed consent, one diagnosis of malignant disease after inclusion, one tissue sample loss, and one competing stroke etiology). In two additional cases, miR levels could not be validly measured at baseline for technical reasons, leaving 21 patients with sCS and 23 patients with aCS. See Table 1 for an overview of demographical and clinical data of these patients. At follow-up, in four additional cases, no miR levels could be measured. These samples failed internal quality control because the CT values of spike-in RNA were significantly too high, indicating insufficient RNA isolation. Data on MES were available for 32 patients. The reasons for missing MES-acquisition were an absent temporal window, emergent surgery, or incompliance leading to discontinuation (11).

**Table 1:** Demographic and clinical characteristics of the study collective (11).

	**sCS (*n* = 21)**	**aCS (*n* = 23)**	***P*-value**
Age (years) [median (Q1–Q3)]	68 (63–74)	70 (60–76)	0.340
Female (%)	3 (14)	8 (35)	0.169
ESRS at baseline [median (Q1–Q3)]	3 (2–4)	4 (4–5)	0.004
Hypertension (%)	16 (76)	20 (87)	0.448
Diabetes mellitus (%)	5 (24)	8 (35)	0.518
Dyslipoproteinemia (%)	17 (81)	18 (78)	0.999
Peripheral artery disease (%)	2 (10)	7 (30)	0.137
Nicotine abuse (%)	16 (80)	18 (78)	0.999
Alcohol abuse (%)	4 (19)	5 (22)	0.999
Coronary artery disease (%)	2 (10)	11 (48)	0.008
Previous myocardial infarction (%)	1 (5)	7 (30)	0.048
Atrial fibrillation (%)	0 (0)	2 (9)	0.489
Previous stroke (%)	3 (14)	5 (22)	0.701
Antithrombotic pretreatment (%)	9 (43)	22 (96)	<0.001
Statin pretreatment (%)	8 (38)	18 (78)	0.013
Maximum NIHSS before inclusion (Q1–Q3)	0 (0–2)	N.A.	N.A.
mRS follow up [median (Q1–Q3)]	0 (0–1)	0 (0–0)	0.638
Grade of stenosis			0.043
Moderate	1 (5)	0 (0)	
Severe	10 (48)	19 (83)	
Highly severe	10 (48)	4 (17)	
Time from onset to blood collection (days) [median (Q1–Q3)]	7 (3–12)	N.A.	N.A.
BMI (kg/m^2^) [median (Q1–Q3)]	27.6 (24.2–29.4)	29.0 (26.2–31.1)	0.097

*BMI, body mass index; ESRS, Essen Stroke Risk Score; mRS, modified Rankin Scale; N.A., not applicable; NIHSS, National Institutes of Health Stroke Scale*.

In patients with sCS, the time from symptom onset to blood collection was calculated as 7 days in median. Of note, vascular risk factors were more frequent in the aCS than in sCS group.

As reported previously, neither the count of MES per hour [median = 0 (IQR: 1) vs. median = 0 (IQR: 1); *p* = 0.766] nor the histological sum score [median = 2 (IQR: 2.5) vs. median = 2 (IQR: 2); *p* = 0.517] differed between the patients with sCS and aCS (11).

**Figure 1:**
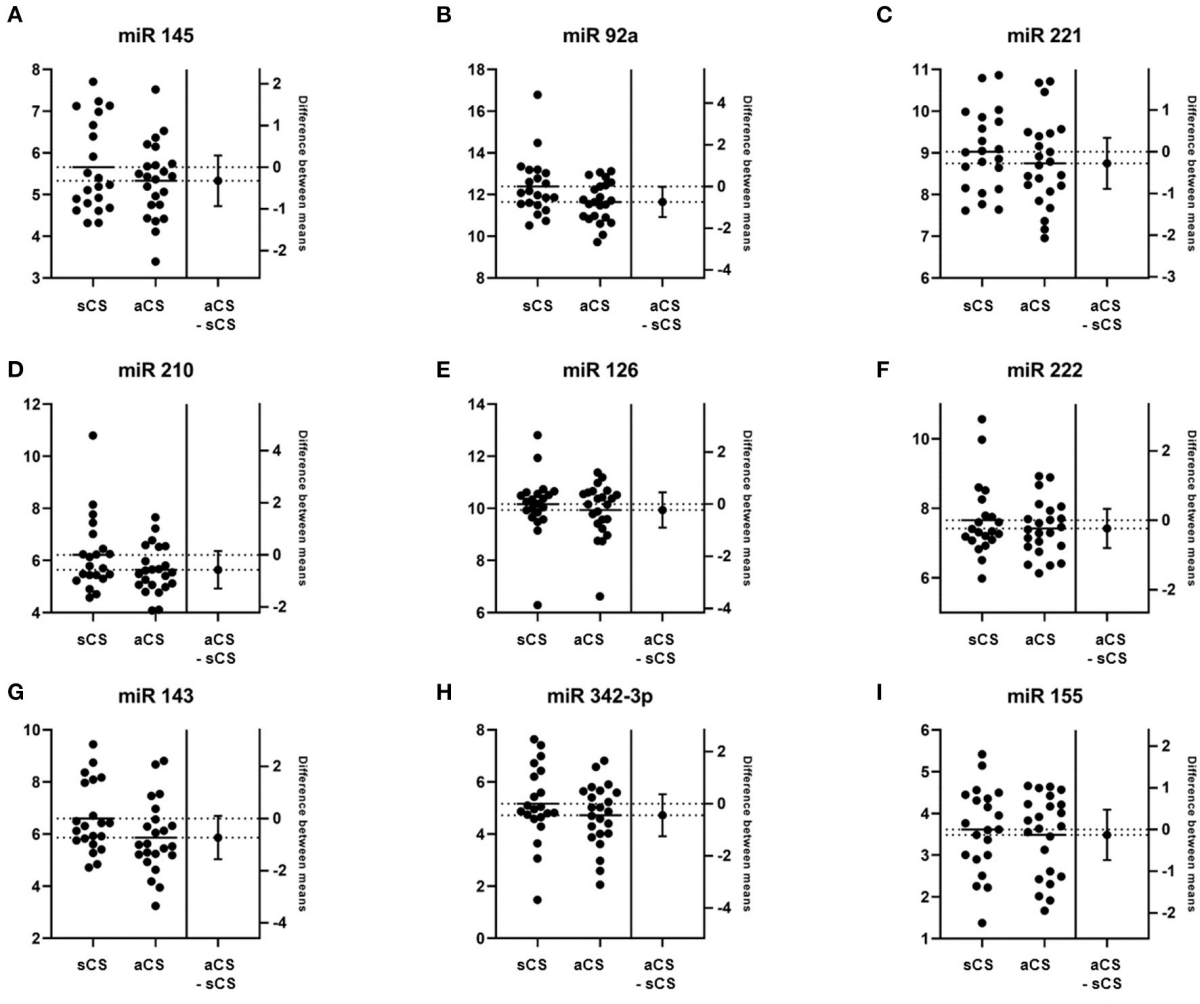
Estimation plots depicting group comparisons of microRNA (miR) levels between sCS and aCS at baseline and the corresponding difference between means (aCS-sCS). **(A)** miR 145, *p* = 0.287; **(B)** miR 92a, *p* = 0.046; **(C)** miR 221, *p* = 0.361; **(D)** miR 210, *p* = 0.235; **(E)** miR 126, *p* = 0.647; **(F)** miR 222, *p* = 0.647; **(G)** miR 143, *p* = 0.081; **(H)** miR 342-3p, *p* = 0.279; **(I)** miR 155, *p* = 0.664.

The mean differences of miRs between groups were overall marginal at baseline, with the relatively largest effects observed for miR-92a, miR-210, miR-145, and miR-143, with best evidence for a difference concerning miR-92a. See Figure 1 for baseline differences of miR levels between the patients with sCS and aCS.

**Figure 2:**
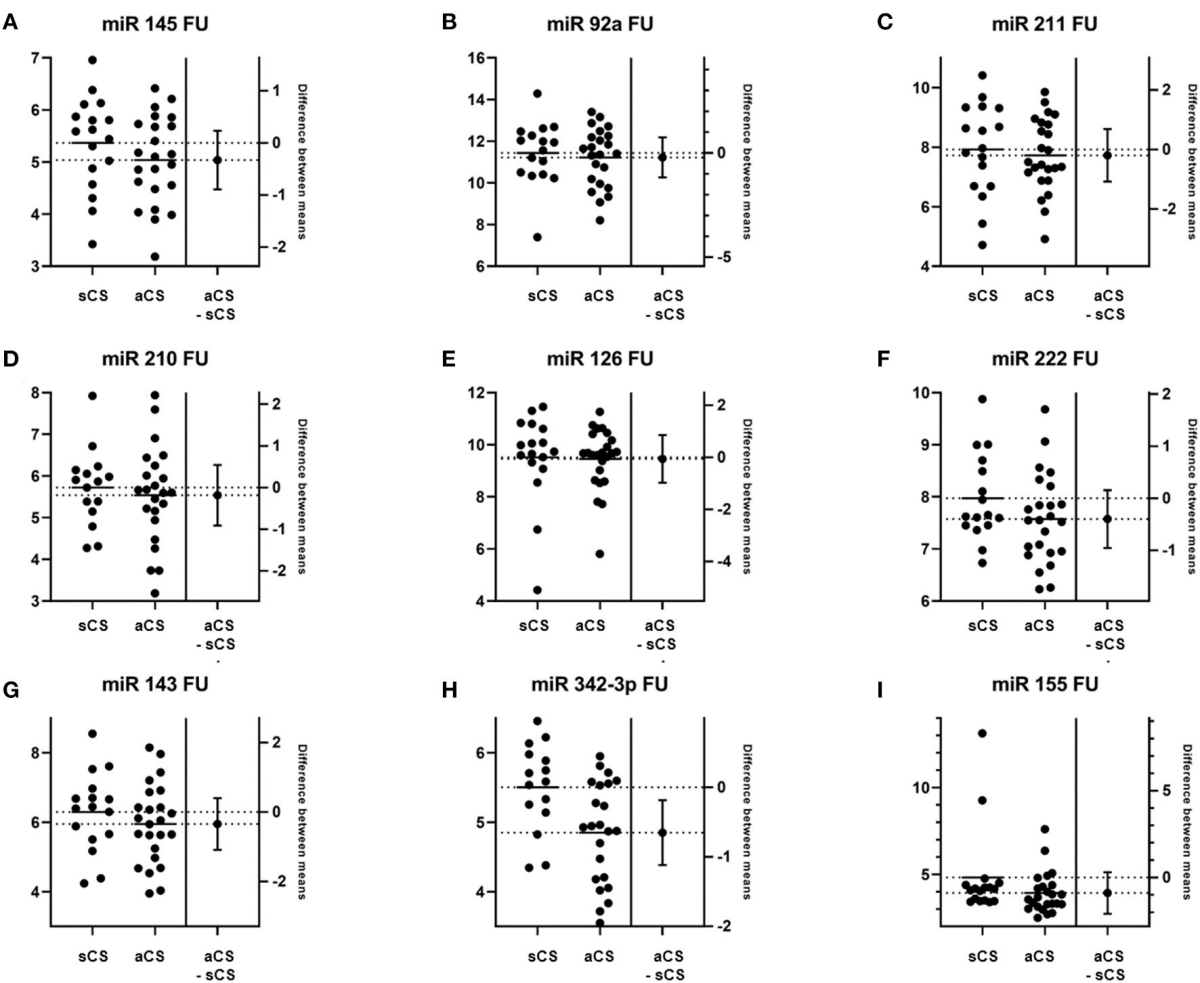
Estimation plots depicting group comparisons of miR levels between sCS and aCS at follow-up (FU) and the corresponding difference between means (aCS-sCS). **(A)** miR 145, *p* = 0.241; **(B)** miR 92a, *p* = 0.655; **(C)** miR 221, *p* = 0.656; **(D)** miR 210, *p* = 0.610; **(E)** miR 126, *p* = 0.525; **(F)** miR 222, *p* = 0.154; **(G)** miR 143, *p* = 0.358; **(H)** miR 342-3p, *p* = 0.007; **(I)** miR 155, *p* = 0.081.

At 90-day follow-up, the mean differences, if present, again were modest. Now, miR-342-3p, miR-222, miR-155, and miR-145 were measured with somewhat higher levels in the sCS than in the aCS group (see Figure 2).

**Figure 3:**
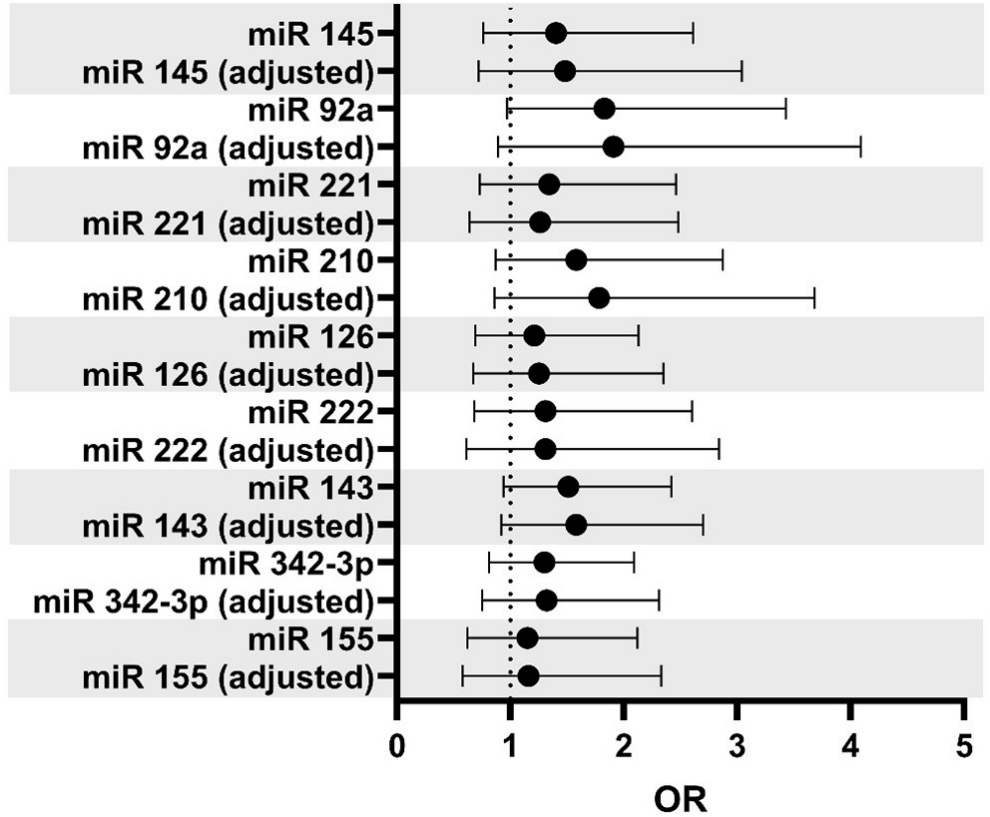
Odds ratios (OR) per log-step increase of miR levels deriving from unadjusted and adjusted logistic regression models with study group as dependent variable. Multivariable regression models were adjusting for the Essen Stroke Risk Score (ESRS) and prevalent statin use. OR >1 indicates higher odds for symptomatic state.

Figure 3 illustrates the results of unadjusted or adjusted logistic regression analyses (adjustment for ESRS and prevalent use of statins). Of note, in both the adjusted and unadjusted analyses, the odds for a symptomatic state was estimated to be slightly higher with higher miR levels, especially regarding miR-92a and miR-210. After adjustment for confounders, the effect estimates were basically unchanged. However, statistical precision of the according effect size estimate was low in each case.

No relevant correlations between baseline miR levels and the ESRS were found within the whole study population or within asymptomatic patients. However, in sCS, there was a positive correlation of miR-143 with the ESRS (Spearman's ρ = 0.449, *p* = 0.041; Supplementary Figure 1).

No significant correlation could be observed between histological sum score and miRs, neither in the whole study group nor in subgroups. However, a trend towards stronger correlation of biomarkers with the histological sum score could be observed within the sCS group (Supplementary Figure 1).

A comparison of patients with and without MES did not reveal relevant miR level differences, neither in the whole study group nor after restriction to the patients with aCS.

## Discussion

Various studies investigated the differences of circulating miR levels in patients with manifest atherosclerosis in comparison to patients without vascular diseases. In this study, on the contrary, we investigated specific circulating miRs in relation to symptomatic state as well as plaque vulnerability of carotid stenosis in patients with manifest atherosclerotic disease. In a previous study of this patient collective, we were able to show distinct differences in cytokine concentrations between sCS and aCS, implying that the sample size was sufficient to detect the relevant blood-based biomarker differences (11). However, we found no relevant group differences in this work and only slightly higher miR levels in sCS than aCS regarding miR-92a, miR-210, miR-145, and miR-143 with best evidence for a modest association of miR-92a with symptomatic state. Patients with aCS revealed more vascular risk factors than patients with sCS, which may be due to more extensive work-up for cardiovascular disorders in this group. In the analysis of biomarkers, one might assume negative confounding resulting from this—however, the effect estimates remained largely the same also after adjustment for the ESRS.

miR-92a was previously shown to play a role in vascular disorders and, more precisely, in endothelial pathology. Bonauer et al. revealed that a therapy targeted against miR-92a might lead to better vascular healing, whereas overexpression leads to damage in ischemic diseases (13). Another study by Loyer et al. demonstrated that an upregulation of miR-92a by oxidized low-density lipoproteins leads to endothelial activation and a stronger progression of atherosclerotic lesions (14). Moreover, in a murine model of myocardial infarction, inhibition of miR-92a resulted in increased endothelial cell autophagy, which was associated with reduced tissue damage and improved survival, making it an interesting target in vulnerable states of atherosclerosis (15). In accordance, miR-92a expression was reported to be higher in patients who suffered from acute myocardial infarction than patients with stable coronary artery disease (16). Others showed a miR-92a increase in patients with aCS vs. healthy controls (17).

miR-210 was previously found to be downregulated in unstable carotid plaque specimens and may promote smooth muscle cell survival (18). Whether a subtle systemic increase in miR-210 in sCS, as shown here, might represent a regulatory response to the acute event needs to be clarified by further investigations.

The miR cluster of miR-143 and miR-145 is crucial in the interaction between smooth muscle cells and endothelial cells (19) and, thus, was considered as potential biomarker in atherosclerotic disease (20). In this study, however, we could not prove the hypothesis that these markers were discriminative between sCS and aCS.

We also investigated whether circulating miR levels were stable over time, hypothesizing that these may reflect a distinct systemic pro-atherogenic environment. At 90-day follow-up, however, the described group differences were even more attenuated, and other markers showed slight differences instead. While it is unlikely that surgery would have an impact on biomarker levels at 90 days, it could be argued that the treatment of a significant atherosclerotic lesion could have a corresponding impact. However, the changes presented here indicate a rather random pattern.

With regard to characteristics of plaque vulnerability and MES as indicators of high-risk carotid stenosis, we were also unable to confirm our hypothesis. However, the finding that possible associations of biomarkers with vascular risk factors as well as vulnerability criteria appear to be accentuated in patients with sCS is consistent with the previously described mechanisms.

There are several limitations to this work. Although other studies investigating miR expression were of comparable or smaller size, the sample size of this investigation is still limited. Due to the study design, biomarker measurements were performed after the embolic event in sCS group, and therefore no predictive value of the biomarkers discussed can be evaluated. In order to minimize the potential effect of cerebral tissue damage, we ensured to include sCS patients with transient ischemia or only small cerebral tissue damage. Indeed, the median NIHSS was 0 in the sCS group. However, longitudinal cohort studies of aCS patients with observation of incident cerebrovascular events would be desirable. In addition, we had to restrict the analysis to certain biomarkers that we considered particularly relevant to the question and cannot rule out the possibility that there may be other markers that would be of relevance. For example, miRs that are associated with fibrosis and closely related to heart diseases, such as miR-21 or the miR-29 cluster, were not investigated due to the scope of this study. Moreover, intraindividual comparative measurements of circulating and tissue-based miRs would be of particular interest in future studies.

In conclusion, this study does not provide evidence for strong associations between particular circulating miRs and symptomatic state in a collective of comprehensively characterized patients with carotid stenosis. No relation of miRs with features of plaque vulnerability was identified. Further work is needed to elucidate the role of circulating miRs as targets in advanced carotid atherosclerosis.

## Data Availability Statement

The raw data supporting the conclusions of this article will be made available by the authors, without undue reservation.

## Ethics Statement

The studies involving human participants were reviewed and approved by Ethics Committee at Hannover Medical School. The patients/participants provided their written informed consent to participate in this study.

## Author Contributions

GMG and AAD conceived the study, analyzed and interpreted data, and wrote the first draft of the manuscript. GMG, RDS, MMG, RS, MW, CS, and KW contributed to the recruitment of patients and interpretation of clinical data. LN, DDJ, and MPK performed the histological analysis and interpretation. AAD, CB, JB, and TT contributed to biomarker measurements, analysis, and interpretation. All authors read and approved the final version of the manuscript.

## Funding

This work was supported by PRACTIS—Clinician Scientist Program of Hannover Medical School, funded by the German Research Foundation (Grant number: DFG, ME 3696/3-1) (GMG and AAD) and by a German Research Foundation grant to DDJ and LN (KFO 311; project Z2).

## Conflict of Interest

TT is founder and shareholder of Cardior Pharmaceuticals GmbH and has filed and licensed patents about non-coding RNAs (outside of this study). The remaining authors declare that the research was conducted in the absence of any commercial or financial relationships that could be construed as a potential conflict of interest.

## Publisher's Note

All claims expressed in this article are solely those of the authors and do not necessarily represent those of their affiliated organizations, or those of the publisher, the editors and the reviewers. Any product that may be evaluated in this article, or claim that may be made by its manufacturer, is not guaranteed or endorsed by the publisher.
