# Supplementary material for: Circulating microRNAs in Symptomatic and Asymptomatic Carotid Stenosis
Source: Front Neurol. 2021 Nov 24;12:755827. doi: 10.3389/fneur.2021.755827 (PMC8651616; doi:10.3389/fneur.2021.755827)
Supplement: Supplementary file 1 [file Image_1.pdf]

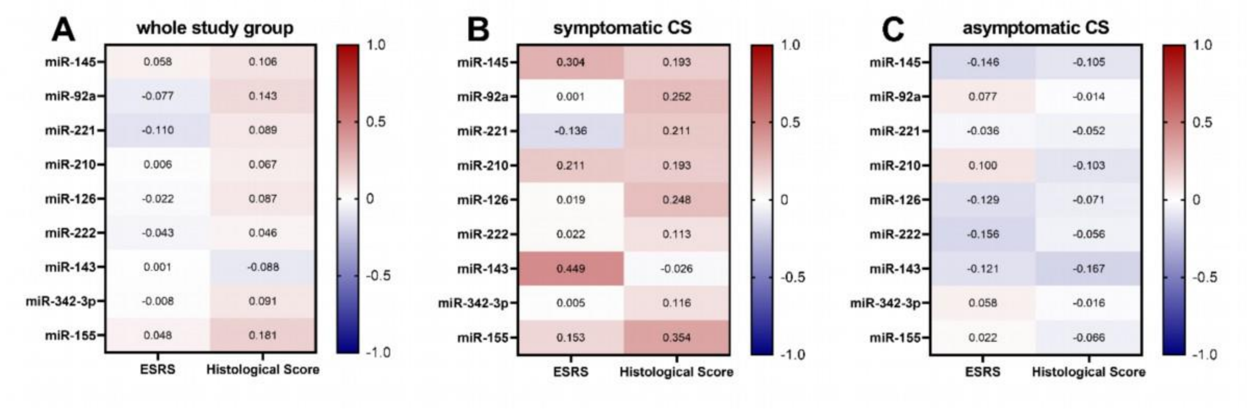

Supplemental Figure 1: Heatmaps of correlations between microRNA levels and ESRS and histological sum score in the whole study group (A), sCS patients (B), and aCS patients (C). Values refer to Spearman's correlation index.
